# Investigations on the Grape Leafhopper *Erasmoneura vulnerata* in North-Eastern Italy

**DOI:** 10.3390/insects10020044

**Published:** 2019-02-01

**Authors:** Carlo Duso, Renzo Moret, Alessandro Manera, Dario Berto, Diego Fornasiero, Gaia Marchegiani, Alberto Pozzebon

**Affiliations:** Department of Agronomy, Food, Natural resources, Animals and Environment, University of Padova, Viale dell’Università 16, Agripolis, Legnaro, 35020 Padova, Italy; carlo.duso@unipd.it (C.D.); renzo_moret@alice.it (R.M.); alessandro@vinimanera.com (A.M.); dario.berto2@hotmail.com (D.B.); diego281m@hotmail.it (D.F.); marchegianigaia@gmail.com (G.M.)

**Keywords:** Cicadellidae, grape, seasonal abundance, development, phenology

## Abstract

The leafhopper *Erasmoneura vulnerata* (Fitch) (Hemiptera: Cicadellidae) is native of Northern and Central America where it occurs on wild and cultivated grapes as well as on a number of secondary hosts. This species was recorded for the first time in Europe (Italy, Veneto region) in 2004. Since then it has spread over Northern Italy and Slovenia. Studies on the biology of *E. vulnerata* in America are limited and thus its phenology was investigated on *Vitis labrusca* L. and *Vitis vinifera* L. plants under field and semi-field conditions. These observations suggest that *E. vulnerata* can complete 2–3 generations per year. The development of *E. vulnerata* from first instar nymphs to adults was studied under controlled conditions (about 23 °C). Developmental times lasted from 16.1 days in July–August to 19.5 days in September, and this variability was probably due to grape cultivar and plant susceptibility. Data were consistent with the number of generations previously reported. *Erasmoneura vulnerata* was more abundant on vines close to overwintering sites than on those located 100–250 m from these sites and contiguous to commercial vineyards.

## 1. Introduction

*Erasmoneura vulnerata* (Fitch) is a leafhopper widely distributed in the USA, Canada and Mexico [1,2,3,4,5]. Its host range comprises primarily North-American grape species as well as *Vitis vinifera* L. (various cultivars), and a number of secondary hosts, mainly *Parthenocissus quinquefolia* (L.) Planch. [6]. Early literature reports *E. vulnerata* as an important pest of cultivated grapes and Virginia creeper [7,8] but more recent studies led us to suspect that this a secondary pest in commercial vineyards [9,10,11].

The life history and behaviour of *E. vulnerata* in America have been little explored. Females oviposit in the vascular bundles of the midrib in contrast to *Erythroneura* spp. which oviposit within the leaf mesophyll [10]. Adults and nymphs feed on the mesophyll which causes pale speckled areas sometimes covering the entire leaf surface; at the same time they produce frass that can affect fruit quality. Intense feeding can cause curled and scorched leaves, and sometimes premature leaf fall. These symptoms were observed in two experimental vineyards in Western Colorado where *E. vulnerata* was associated with the more abundant *Erythroneura ziczac* (Walsh) [11]. Overwintered adults of *E. vulnerata* colonised these vineyards in May, moving from the periphery. *E. vulnerata* completed two generations because nymphal densities peaked on leaves in June and August [11].

In 2004, *E. vulnerata* was first detected in Europe, in the Veneto and Friuli-Venezia Giulia regions, Northeastern Italy [12]. During 2006 and 2007, this species was found in various sites in Veneto, Friuli-Venezia Giulia (close to the border with Slovenia), Emilia-Romagna and Trentino-Alto Adige regions [13]. In 2010, *E. vulnerata* was recorded in Slovenia [14]. In these surveys, moderate to high population densities were recorded on unsprayed vines of *Vitis labrusca* L. and French hybrids as well as on *Parthenocissus* spp. growing in gardens. In contrast, the occurrence of *E. vulnerata* in commercial vineyards (including organic ones) was negligible. Vineyards located in this area are subject to mandatory insecticide programs to control *Scaphoideus titanus* Ball, vector of *Candidatus Phytoplasma vitis*, which causes “Flavescence dorée” disease [15,16].

In this paper we report the results of field studies investigating *E. vulnerata* phenology. At the same time we conducted laboratory studies to evaluate generational developmental times of *E. vulnerata*. The most common leafhoppers infesting vineyards in Europe (e.g., *Empoasca vitis* Göthe and *Zygina rhamni* Ferrari) overwinter as adults on trees (e.g., conifers, *Carpinus betulus* L., *Quercus* spp.) contiguous to vineyards. Adult leafhoppers first colonized the vineyard plots close to the overwintering sites then the inner ones [17,18,19]. The role of overwintering sites in affecting *E. vulnerata* seasonal abundance on different grape cultivars was also evaluated.

## 2. Materials and Methods

### 2.1. The Phenology of *Erasmoneura vulnerata* on *Vitis labrusca*

The phenology of *E. vulnerata* was investigated from 2005 to 2007 on large *V. labrusca* (cv. Isabella) plants growing in a garden at Conegliano (Treviso province, Northeastern Italy). In the previous season, hotspots of *E. vulnerata* were detected on these grapevines that were not treated with pesticides. This species was rarely observed in a nearby commercial vineyard where plant protection products (fungicides and insecticides) were routinely applied. Two yellow sticky traps were placed within the grapevine canopy during the growing season to delineate adult flight patterns of the most important leafhopper taxa. Traps were analysed in the laboratory under a dissecting microscope and replaced every 1–2 weeks. A total of 40–100 leaves (depending on the season) were inspected every 1–2 weeks and analysed in the laboratory under a dissecting microscope. Leafhopper individuals were counted considering their identity and age, and subdivided into three categories (I–II instar nymphs, III–V instar nymphs, adults).

### 2.2. The Biology of *E. vulnerata* in Semi-Field Conditions

The biology of *E. vulnerata* was studied in 2008 on potted vines at the farm where this species was detected for the first time in Europe (Castelfranco Veneto, Treviso province, see Duso et al. [12]). A total of 11 potted *V. vinifera* cv. Cabernet Franc and 11 potted *V. labrusca* cv. Isabella vines were used in a completely randomized block experiment. Each vine was placed in a cage consisting of a cylindrically shaped metal structure covered by a sleeve closed at both ends. Potted vines were placed under a roof to reduce the impact of rain and thus of downy mildew infections. At the beginning of the study (11 July), five females and five males of *E. vulnerata* were introduced into each grapevine cage. Their sex had been checked under a dissecting microscope. The adults were kept inside the cages for one week and subsequently removed (18 July) using an aspirator. The experiment was completed when all nymphs were expected to have molted to adulthood. Vines were inspected every 2–4 days to assess the abundance of different active forms (I–II instar nymphs, III–V instar nymphs and adults).

### 2.3. Nymphal Development

Nymphal development of *E. vulnerata* was investigated on caged potted vines in the laboratory (23 ± 2 °C, 70% R.H.). Cages were similar to those previously described. At the beginning of this experiment (1st day), one female and one male of *E. vulnerata* were confined in each grapevine cage. The adults were kept inside the cages for up to 7 d and were subsequently removed. The experiment was completed when all nymphs were expected to have molted to adulthood. The abundance of different motile stages (nymphs and adults) was assessed every 2 days. The experiment was repeated in September 2010 (cvs. Carménère and Chardonnay) and in July-August of 2011 (cv. Merlot). Results are restricted to leafhopper females.

### 2.4. The Effect of Overwintering Sites on *E. vulnerata* Seasonal Abundance

At the above-mentioned farm, we identified three areas (A1, B1, C1) to evaluate the effect of distance from potential overwintering sites on the seasonal abundance of *E. vulnerata*. Area A1 was characterized by the presence of rural buildings and alternative host plants (e.g., *Parthenocissus* spp.); area B1 was 120 m from A1 and was characterized by the presence of annual crops and isolated grapevines; area C1 was located 250 m from A1, between two commercial vineyards but approximately 40 m from them to minimize insecticide drift effects. A total of 30 potted vines belonging to two cultivars commonly cultivated in this area, i.e., Cabernet Sauvignon and Refosco dal peduncolo rosso (Refosco p.r.), were placed in each area along three transects (10 plants for each cultivar per transect per area). The distance between each transect was about 20 m. Prior to the trials (three days) potted vines were sprayed with pyrethrins (Piresan plus^®^, Intrachem Bio Italia S.p.A., Grassobbio, Italy) to eliminate naturally occurring leafhoppers. Observations started on 12 July and ended on 10 October 2008; they were carried out every week on 4 ad hoc selected leaves per vine with the help of a magnifying glass (×10). During the observation period potted vines were treated three times with fungicides (mancozeb plus dimetomorf, Forum MZ, Basf, Cesano Maderno, Italy) to reduce downy mildew risk and preserve leaf health.

A similar study was planned in 2009 at the same farm, but only two areas were included in the comparison. The first of them (A2) was contiguous to farm facilities and the second (B2), located about 100 m from A2, was close to a commercial vineyard (approximately 40 m). A total of 30 potted vines (15 of cv. Cabernet Sauvignon and 15 of cv. Refosco p.r.) were placed in each area (three transects with five plant per cultivar per each area). The sampling procedure was the same as during the previous season but observations started in May and finished in October. Vines were sprayed with fungicides (mancozeb plus dimetomorf, Forum MZ, Basf) to prevent downy mildew damage.

### 2.5. Statistical Analysis

Data on nymphal (subdivided into I–II instars and III–V instars) and adult abundance obtained from the caged plant experiment (see Section 2.2) were analyzed using a Restricted Maximum Likelihood (REML) repeated measures model with the Proc MIXED of SAS^®^ (ver. 9.4; SAS Institute Inc., Cary, NC, USA). Grape cultivar, time and their interaction were considered as sources of variation and were tested using an F test (α = 0.05). The average numbers of adults and nymphs observed on a plant were considered as response variables with repeated measures made at different times, i.e., sampling dates. Degrees of freedom were estimated using the Kenward–Roger method. First-order autoregressive and spatial power covariance structures for correlating different sampling dates on the same plant were considered for respective evenly and unevenly spaced time intervals. Different transects were considered as random effect term in the models. Differences among treatments were evaluated with a Bonferroni test (α = 0.05) on least square means. Data were checked for normality and thus the numbers of insects were transformed to log (x + 1).

Similar models and statistical methods were used for the analysis of data on the effect of overwintering sites investigated in 2008 and 2009 (see Section 2.4). In these models, area, grape cultivar, time and their interactions were considered explanatory variables and were tested with an F test (*α* = 0.05) on their effect on the total number of *E. vulnerata* individuals observed per leaf. Data were checked for normality assumption and thus the numbers of insects were transformed to log (x + 1).

No statistical analysis was applied to Section 2.1 and Section 2.3 and observational data were obtained.

## 3. Results

### 3.1. The Phenology of *Erasmoneura vulnerata* on *Vitis labrusca*

**Figure 1:**
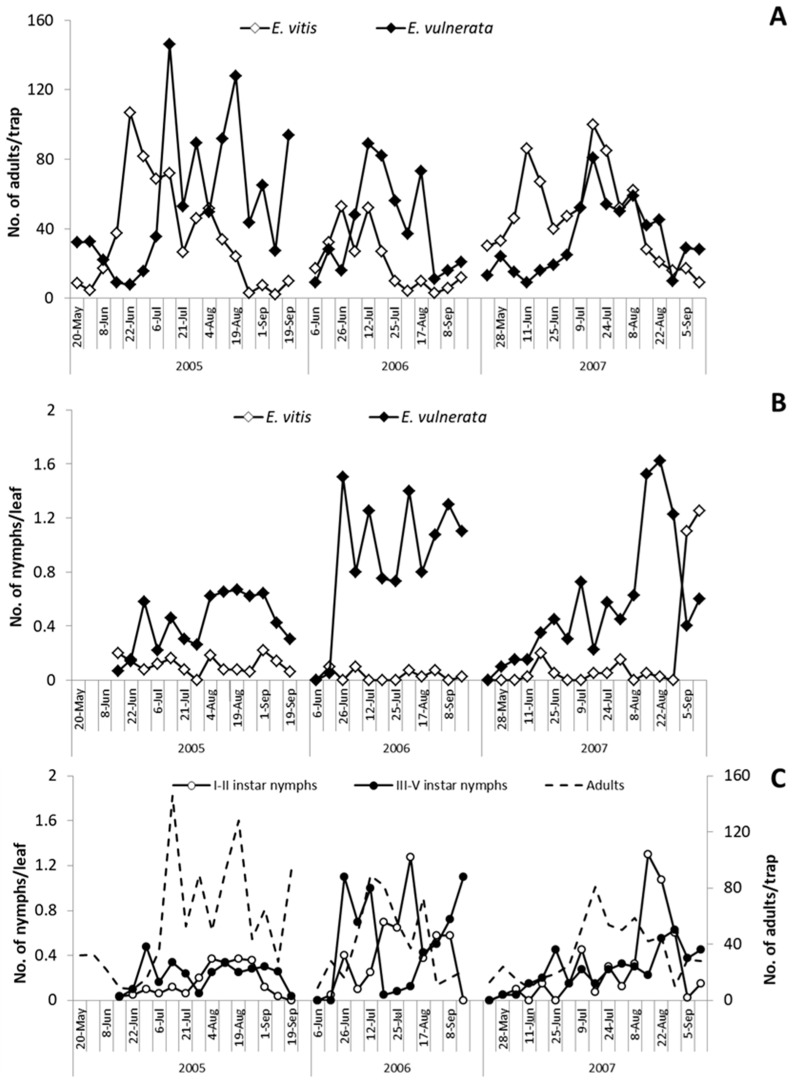
Adult catches (**A**) and nymphs seasonal abundance (**B**) of *Empoasca vitis* and *Erasmoneura vulnerata* on *Vitis labrusca* (cv. Isabella). The phenology of *Erasmoneura vulnerata* on *Vitis labrusca* (cv. Isabella) is reported below (**C**). Adults were counted on yellow sticky traps, while nymphs were counted on leaves during three subsequent seasons.

In the spring of 2005, *E. vulnerata* and *E. vitis* were the most frequent leafhoppers detected on the canopy of *V. labrusca* (Figure 1). Catches of *Z. rhamni* did not exceed 20 adults per trap and they became negligible over the season (data not reported). *Erasmoneura vulnerata* catches were relatively abundant in May and from July to September; those of *E. vitis* were most abundant in June. The analysis of leaf samples showed a clear dominance of *E. vulnerata* nymphs (Figure 1). When sampling started (14 June 2005), III–V instars nymphs (older nymphs) of *E. vulnerata* were observed on leaves, suggesting that this generation started earlier (Figure 1). Nymph densities peaked from late June to mid-July and from early August to early September. Adult catches of *E. vulnerata* showed three major peaks and the first two of them followed those of older nymphs. Data suggest that in 2005, *E. vulnerata* completed at least two generations.

In spring 2006, catches of adult *E. vitis* were higher than those of *E. vulnerata* but the opposite was noticed from July onwards (Figure 1). Regarding nymphs, *E. vulnerata* clearly dominated over *E. vitis* throughout the season (Figure 1). Leaf sampling started in early June when no *E. vulnerata* early instar nymphs were detected. Their densities peaked in late June followed by relatively high adult numbers (Figure 1). Climatic conditions in May were characterized by relatively low temperatures and frequent rainfall. These conditions probably caused a delay in the development of *E. vulnerata* nymphs. Early instar nymph (I–II instars) numbers peaked again from late July onwards while for older nymphs, from August to September. Temperatures were relatively high between mid-June and late July, but August was relatively cold and cool; these conditions probably again delayed nymphal development (Figure 1). Adult catches peaked in early September.

In the first part of the 2007 growing season, *E. vitis* adult catches were higher than those of *E. vulnerata*; later catches of the two species overlapped (Figure 1). However, *E. vulnerata* was frequently dominant over *E. vitis* in terms of nymphs observed on leaves (Figure 1). Regarding *E. vulnerata* phenology, early instar nymphs were detected in late May and their numbers slightly increased in June (Figure 1). Additional peaks were noticed in July and late August. The seasonal abundance of older nymphs was consistent while that of adults did not display clear patterns. Climatic conditions in 2007 were characterized by high temperatures in April. Therefore, nymphs appeared earlier than in previous years. June was rainy and this probably affected the nymph abundance that fluctuated at low population densities. In July, temperatures increased again with positive effects on leafhoppers.

### 3.2. The Biology of *E. vulnerata* under Semi-Field Conditions

**Figure 2:**
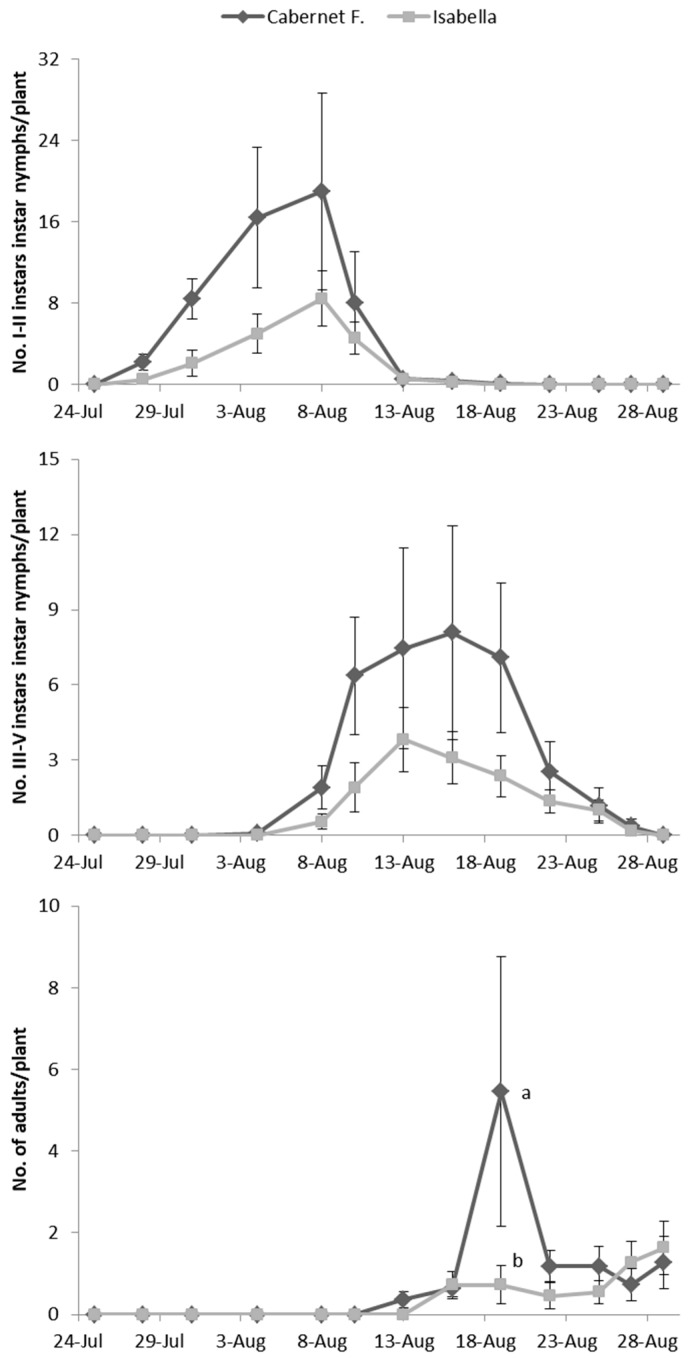
Abundance of *Erasmoneura vulnerata* nymphs and adults observed on caged potted vines (2008). Different letters indicate significant differences based on the Bonferroni test (*p* = 0.05).

*E. vulnerata* adults were confined in cages containing potted vines on 11 July and were removed one week later. Early instar nymphs were first detected on 28 July and most of them from 31 July to 4 August (Figure 2). Older nymphs were recorded from 8 August onwards and their abundance showed a peak between 13–16 August. The first adults emerged on 13 August and the last ones on 29 August. About 50% of adults (86 out of 178) emerged from the 19 to the 22 of August.

No effect of cultivar was found on the abundance of nymphs and adults of *E. vulnerata* on caged plants (F test—I–II instars: *p* = 0.561; III–V instars: *p* = 0.272; adults: *p* = 0.256; Figure 2) while a variation through time was observed (I–II instars: *p* < 0.001; III–V instars: *p* < 0.001; adults: *p* < 0.001; Figure 2). The interaction between time and cultivar was significant for adults but not for nymphs (I–II instars: *p* = 0.354; III–V instars: *p* = 0.065; adults: *p* = 0.006), because higher numbers of adults were recorded on 19 August in Cabernet Franc than on Isabella plants (Figure 2).

### 3.3. Nymphal Development

Laboratory studies on the development of *E. vulnerata* from first instar nymphs to adult females were carried out under the same controlled conditions but in two periods. In September 2010, nymphal development was 19.37 ± 0.49 (mean ± std. err.) and 19.66 ± 0.16 days on cvs. Carménère and Chardonnay, respectively. In the additional experiment on cv. Merlot, carried out in July–August of 2011, nymphal development required 16.1 ± 0.07 days.

### 3.4. The Effect of Overwintering Sites on *E. vulnerata* Population (2008)

Leafhopper numbers reached different densities in the three areas at different distances from potential overwintering sites (F test—*p* < 0.0001) and were more abundant close to rural buildings (0.18 ± 0.01 active forms per leaf) than in the open field (0.07 ± 0.01 active forms per leaf) or further away (0.01 ± 0.01 active forms per leaf) (respectively: t_173_ = 3.56; *p* = 0.001; t_173_ = 5.93; *p* < 0.001). There were no differences between the last two (t_173_ = 1.92; *p* = 0.169). The effects of “time” and of interaction “area × time” were also significant (*p* < 0.001; *p* < 0.001, respectively). In particular, a significant variation in *E. vulnerata* numbers over the season was recorded at the site near buildings (*p* < 0.001), but not in the other ones (C1: *p* = 1; B1: *p* = 1). No effects of cultivar and interaction “cultivar × area” emerged (respectively *p* = 0.410; *p* = 0.259), while a significant effect was found for the interactions “cultivar × time” and “cultivar × area × time” (respectively *p* = 0.003; *p* < 0.001), because in the A1 area, a significant time effect was observed on Cabernet Sauvignon (*p* < 0.001), but not on Refosco p.r. (*p* = 0.8102).

**Figure 3:**
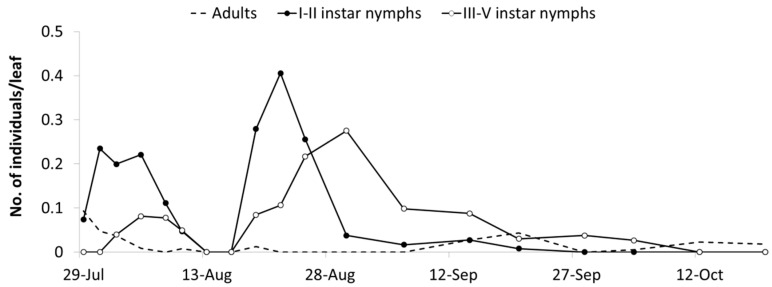
Seasonal abundance of *Erasmoneura vulnerata* nymphs and adults observed on leaves of potted vines placed close to rural buildings (A1 area, 2008).

Observations carried out in the A1 area from mid-July to late September provided additional data on the phenology of *E. vulnerata* (Figure 3). Early instar nymphs occurred first on 28 July and their densities showed two peaks, on 31 July and 22 August, followed by those of older nymphs (4 and 30 August). These data strongly suggest the development of two nymphal generations. The first nymphal generation developed faster than the second one, probably because of different temperature regimes recorded during these phases (average temperature were 24.2 and 20.1 °C, respectively) and plant conditions.

### 3.5. The Effect of Overwintering Sites on *E. vulnerata* Abundance (2009)

*Erasmoneura vulnerata* population densities reached higher densities in A2 (close to rural buildings, 0.54 ± 0.08 active forms per leaf) than in the B2 area (close to commercial vineyards; 0.04 ± 0.01 active forms per leaf) (F test—*p* < 0.001). A significant interaction of “area × cultivar” was found (*p* < 0.001) because in the A2 area, higher densities were reached on cv. Cabernet Sauvignon than on cv. Refosco p.r. (*p* < 0.001), while no differences were observed in the B2 area (*p* = 0.427). A significant variation of *E. vulnerata* numbers was observed in time (*p* < 0.001), and the interaction “area × time” was significant (*p* < 0.001) because variation in time was significantly greater in A2 than in B2.

**Figure 4:**
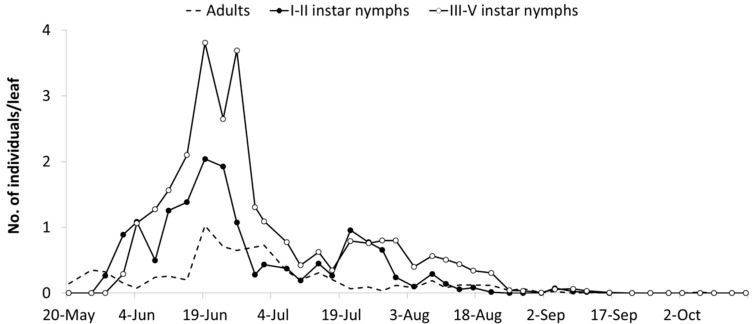
Seasonal abundance of *Erasmoneura vulnerata* nymphs and adults observed on leaves of potted plants placed close to rural buildings (A2 area, 2009).

The abundance of *E. vulnerata* in the A2 area allowed the tracking of the leafhopper phenology in the first part of the growing season. Adults were detected on the first sampling date (20 May). Early instar nymphs were first observed on 25 May and their densities peaked in mid-June (Figure 4). This trend was followed by that of older nymphs and adults. Later leafhopper densities declined. Early instar nymphs increased again in number in the second half of July followed by older nymphs. Adult numbers were lower than expected. In August, leafhopper densities declined further, probably because leaves deteriorated through the prolonged leafhopper feeding.

**Figure 5:**
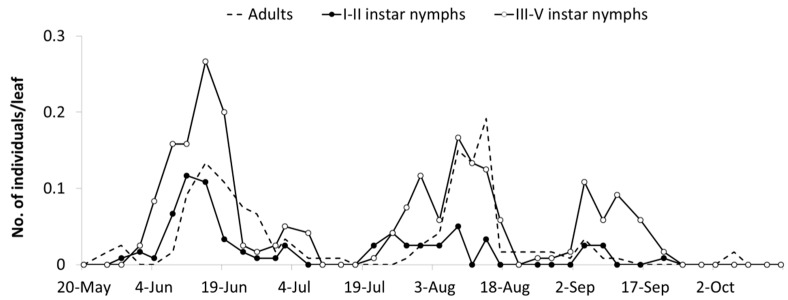
Seasonal abundance of *Erasmoneura vulnerata* nymphs and adults observed on leaves of potted plants placed 100 m far from A2 area (B2 area, 2009).

*E. vulnerata* densities were lower in the B2 than in the A2 area but these records offered a complementary picture of the leafhopper phenology in the 2009 growing season. The first generation was almost synchronized with that observed in area A1, while the second generation was slightly delayed (Figure 5). An additional nymphal peak was recorded in the first half of September, suggesting the development of an additional generation. Climatic conditions during the 2009 summer were characterized by relatively high temperatures between 20 July and 20 August (average temperature 25.6 °C) when the second generation appeared. In the subsequent four weeks, favorable climatic conditions persisted (22 °C as an average).

## 4. Discussion

*Erasmoneura vulnerata* adults colonize vines in the spring, moving from neighboring overwintering sites. The proximity to natural (e.g., conifers) and artificial (rural buildings) overwintering sites was crucial for the establishment of leafhopper populations on vines. Experiments with potted vines showed higher leafhopper densities on our experimental vines close to overwintering sites compared to those further away. The colonization by adults was successful on both *V. labrusca* and *V. vinifera* cultivars.

When observations on *V. labrusca* started at bud break, the first nymphs were detected in late May–early June. Two, sometimes three nymphal peaks were detected over the growing season. Two peaks were recorded in 2005, when mid-summer was characterized by relatively low temperatures (in August, temperatures were frequently 3–4 °C lower than average temperatures) associated with frequent rains. Two peaks were also recorded on potted vines from late July to early October 2008, suggesting that an additional generation could develop in spring. Two to three nymphal peaks were recorded on potted vines in 2009 when observations were prolonged throughout the overall growing season. In this year, two peaks associated with high population densities were detected in the A2 area while three peaks were observed in the B2 area where populations were much lower. We suggest that deteriorated leaf conditions in potted vines did not allow for the development of the third nymphal generation. These observations suggest that during the study period (2005–2009), *E. vulnerata* completed two–three generations per year in North-eastern Italy.

In Colorado, Zimmermann et al. (1996) observed two nymphal peaks (in June and August) and concluded that *E. vulnerata* could complete two generations. It should be mentioned that the *E. vulnerata* population was about 15% of the total leafhopper population in these vineyards, and thus competition with the dominant *E. ziczac* could have influenced the phenology of the first species. When discussing competition with *E. ziczac*, the authors suggest that *E. vulnerata* could maladapt to the dry climate and high altitude of these Western Colorado areas. Interspecific competition and pesticide use clearly had an effect on *E. vulnerata* in Colorado. Therefore, a number of environmental (e.g., climate) and cultural (e.g., the susceptibility of *Vitis* species/cultivars, pesticide use) factors could explain the different population dynamics observed in the original (North America) vs. the newly invaded area (Europe). Detailed studies on *E. vulnerata* biology under controlled conditions are needed to support phenological data. Results of the laboratory study on *E. vulnerata* development can contribute to a first evaluation of the demographic parameters of this species. Developmental times from first instar nymphs to adult females lasted from 16.1 ± 0.16 days in July–August to 19.7 ± 0.49 days in September. Experiments were carried out under controlled conditions (about 23 °C) and thus differences among data series could be due to the different susceptibility of cultivars. Studies carried out on potted vines showed the higher susceptibility of Cabernet Franc compared to Isabella and of Cabernet Sauvignon compared to Refosco p.r. Among factors involved in this phenomenon, leaf hairiness could be involved [9] as reported for other leafhoppers [20,21]. It should be emphasized that Refosco p.r. leaves are much hairier than Cabernet Sauvignon leaves [22]. Leaf pubescence of Isabella leaves is also a typical character of this cultivar. On the other hand, differences in nymphal development could be affected by the season. We suggest that vines used in the experiment of September (late season) were less susceptible to leafhoppers than those used in July–August.

Studies on related leafhoppers in North America, i.e., *E. ziczac* and *E. elegantula* Osborn, offer an interesting comparison with results obtained on *E. vulnerata* [23]. Under controlled conditions, nymphal development of *E. ziczac* lasted from 16.9 (at 25 °C) to 24.3 (at 21 °C) days while that of *E. elegantula* ranged from 18 (at 25 °C) to 26.5 (at 21 °C) days. These times are slightly longer than those reported for *E. vulnerata* in July–August (16.1 days at 23 °C), although different rearing methods were used in the two studies. Egg duration of *E. ziczac* lasted from 11.2 (25 °C) to 18.9 (20 °C) days. Therefore, the overall development of *E. ziczac* in the laboratory required approximately 28 days at 25 °C and 43 days at 20–21°C. We did not measure the developmental times of *E. vulnerata* eggs but we can estimate them as well as the overall leafhopper development. In the first semi-field study performed in the present work (2008 study), most early instar nymphs were detected from 31 July to 4 August. Considering 15 July as an average date for *E. vulnerata* oviposition, we estimate that the duration of egg development ranged from 16 to 20 days, and overall development for adults required 35–38 days. It should be stressed that the average temperature during the semi-field study (23.2 °C) was similar to that adopted in the laboratory study (23 ± 2 °C) where nymphal development required 16.1 days. Therefore, laboratory data were consistent with those obtained on caged vines under semi-field conditions. Using uncaged potted vines (see A1 area, 2008), the development of nymphs in July appeared to be faster than that observed on caged potted vines. This study started on 12 July but potted vines were treated with pyrethrins which have a residual activity of about 2–3 days. Therefore, we can assume that oviposition in uncaged potted vines probably started on 15 July. Early instar nymphs occurred first on 29 July as in the study with caged potted vines. However, about half of the older nymphs were detected between 5–8 August while on caged potted vines, one week later. The best conditions of uncaged potted vines compared to caged potted vines and the higher temperature they experienced could have influenced the different patterns observed in the two studies.

## 5. Conclusions

In conclusion, estimated generational times of *E. vulnerata* are comparable with those calculated for *E. ziczac* suggesting the potential of the former species to become a pest in southern Europe. Recently, local outbreaks have been detected in North-Italian vineyards and this phenomenon has been associated with reduced susceptibility toward pesticides commonly used in viticulture, in particular, organophosphates [24]. At the same time, investigations should shed light on relationships between leafhoppers and their natural enemies, especially the Hymenoptera Mymaridae. The first observations revealed a low impact of these egg parasitoids on *E. vulnerata* [25]. Nevertheless, our study shows that *E. vulnerata* can complete two-three generations per year and successfully colonize several European grapevine cultivars. Further studies on the pest bio-ecology together with an assessment of (direct and indirect) pesticide effects are needed to develop effective ad hoc IPM strategies.
